# Influence of Post-Washing Time and Build Orientation on Mechanical Properties and Biocompatibility of Additively Manufactured Permanent Dental Resin Material

**DOI:** 10.3390/polym17192694

**Published:** 2025-10-05

**Authors:** Pei-Wen Peng, Jia-Syuan Chou, Le-Xin Chen, Po-En Chuang, Hidekazu Takahashi, Min-Chieh Hsieh, Wei-Fang Lee

**Affiliations:** 1 School of Dental Technology, Taipei Medical University, Taipei 11031, Taiwan; apon@tmu.edu.tw (P.-W.P.); b210110014@tmu.edu.tw (J.-S.C.); b210110019@tmu.edu.tw (L.-X.C.); b210110050@tmu.edu.tw (P.-E.C.); 2 Department of Oral Biomedical Engineering, Institute of Science Tokyo (Science Tokyo), Tokyo 152-8550, Japan; takahashi.bmoe@tmd.ac.jp; 3 Department of Prosthodontics, Shin Kong Wu Ho-Su Memorial Hospital, Taipei 11008, Taiwan

**Keywords:** digital light processing, DLP, permanent crown, post-washing time, build orientation

## Abstract

Background: Digital light processing (DLP) is widely used in permanent dental restorations for its precision and efficiency, yet the effects of build orientation and post-washing time on resin properties remain unclear. This study aims to investigate the factors that impact the performance and biocompatibility of DLP-printed dental resins. Methods: Specimens were additively manufactured using permanent dental resin at 0°, 15°, and 90° orientations and post-washed for 90, 120, or 150 s. Evaluated properties included dimensional accuracy, hardness, flexural strength and modulus, degree of conversion, water sorption/solubility, and cytotoxicity. Results: Build orientation and post-washing time significantly affected dimensional accuracy, with thickness showing the least deviation. Flexural strength (*p* < 0.001) and modulus (*p* < 0.01) were highest at the 0° orientation. Post-washing for 90 s led to the greatest water absorption, while solubility remained unaffected. Cell viability increased with longer post-washing times, peaking at 150 s, with significant effects observed on days 5 and 7 (*p* < 0.05). Conclusions: Build orientation significantly affected dimensional accuracy and flexural strength, while post-washing time had minimal impact on physical properties. Notably, extended post-washing improved cell viability and reduced cytotoxicity, indicating its potential to enhance the clinical biocompatibility of DLP-fabricated dental resin.

## 1. Introduction

Dental restorations have been widely fabricated using computer-aided design/computer-aided manufacturing (CAD/CAM) techniques, encompassing subtractive and additive manufacturing methods [1,2,3,4]. These cutting-edge technologies have gradually supplanted conventional fabrication practices in numerous dental applications due to their ease of learning and rapid, high-quality fabrication capabilities. Additive manufacturing, commonly known as 3D printing, creates objects layer by layer, minimizing material waste and eliminating the need for milling burs. The expanding range of 3D printing technologies, including vat polymerization methods like digital light processing (DLP) and stereolithography apparatus (SLA), have been applied to fabricate surgical guides, fixed dental prostheses, and complete dentures [5].

Currently, additively manufactured polymer-based dental crowns and bridges are popularly applied in clinical dentistry with high replicable accuracy and less chairside work, and they must meet specific requirements to ensure durability, functionality, and biocompatibility [6]. Clinical occlusal forces typically range 12–90 N, with occasional maximum forces reaching up to 909 N in posterior areas [7]. Therefore, one crucial aspect is to demonstrate sufficient mechanical performance to withstand daily masticatory forces and opposing tooth wear without fracturing or breaking during extended use [8]. Another crucial aspect is of a material’s surface characteristics, for which the materials and structures must possess biocompatibility and exhibit clinically acceptable behaviors of water sorption (Wsp) and solubility (Wsl) [9]. Due to the susceptibility of additive manufacturing polymer-based permanent restorations to dissolve or disintegrate when exposed to saliva or oral fluids in the oral environment, chemical stability is essential for maintaining oral health and protecting the remaining tooth structure. A permanent restoration prevents direct contact with the oral environment, which has a significant impact on the quality of a patient’s life [10].

In the DLP and SLA fabricating processes, a liquid photopolymer is exposed to a light source, causing the resin to crosslink and solidify layer-by-layer until the object is fully completed [11]. This layer-based manufacturing process contributes to the anisotropic properties of additively manufactured objects. Achieving clinically acceptable margins and optimizing the mechanical properties of objects rely heavily on identifying appropriate fabricating parameters. These parameters include build orientation, layer thickness, light exposure time, post-processing techniques, and other relevant factors [12]. Build orientation and layer thickness are critical factors influencing the surface roughness, geometric accuracy, and overall product quality of additively manufactured objects [13,14,15,16,17]. DLP-fabricated objects require specific post-processing, including cleaning excessive resin, removing support structures, and undergoing a curing process to increase the conversion of the photopolymer [18]. Post-curing strategies and cleaning methods were reported to impact the mechanical stability, two-body wear, and fracture load results of printed fixed dental prostheses [8,11].

Continuous advancements in photoactivated polymers have opened up new possibilities for utilizing them to produce permanent prostheses [19,20]. However, the evaluation of additively manufactured permanent prostheses remains relatively limited in existing studies. Additionally, effects of an appropriate post-washing time on a fabricated resin’s surface characteristics, mechanical performance, and biocompatibility have received little attention. In the current study, we investigated impacts of different build orientations and cleaning times on characteristics and mechanical properties of DLP-fabricated permanent resin specimens intended for long-term clinical use. The properties examined encompassed the dimensional accuracy, flexural strength, flexural modulus, Vickers hardness (VH), degree of conversion (DC), water sorption and solubility, and biocompatibility. The null hypothesis posited that the build orientation and post-washing time would not influence the characteristics, mechanical properties, accuracy, or biocompatibility of DLP-fabricated permanent resins.

## 2. Materials and Methods

In this study, mechanical properties of DLP-fabricated permanent resin specimens were initially tested to determine the optimal printing orientation. Subsequently, assessments were performed for the DC, water sorption and solubility, and cytotoxicity.

### 2.1. 3D CAD and Specimen Preparation

The material used in the present study was a polymer-based resin (Detax Freeprint Crown composite, A2, DETAX, lot no. 260101, Ettlingen, Germany) for DLP-fabricated permanent single crowns, denture teeth, and long-term temporary bridges. Base on the safety data sheet provided by the manufacturer [21], this material is primarily composed of multifunctional dimethacrylate cross-linkers (e.g., urethane dimethacrylate derivatives), together with lower molecular weight monomers such as 1,6-hexanediol dimethacrylate and hydroxypropyl methacrylate. According to the modified ISO 10477:2020 standard [22], the study utilized two types of specimens designed using CAD software (Solidworks 2016, Dassault system, Waltham, MA, USA), including bar-shaped specimens measuring 25 × 2.5 × 2 mm and disk-shaped specimens with a 10-mm diameter and a 2-mm thickness.

**Figure 1:**
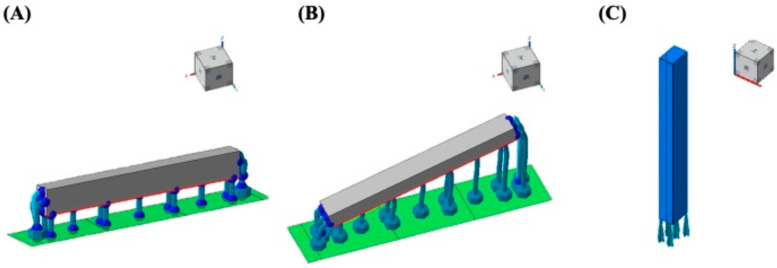
Specimens were fabricated on a build platform with orientations of (**A**) 0°, (**B**) 15°, and (**C**) 90°. In the 0° orientation, samples were printed flat on the build platform (XY plane = length × width, Z = thickness). The 90° orientation corresponds to samples printed vertically, with the length axis aligned to the build direction (*Z*-axis). The 15° orientation indicates that the long axis of the specimen was tilted 15° relative to the XY plane.

The designed specimens were saved in STL file format and subsequently imported into built-in slicing software. Automatic support structures were generated for each specimen. Then specimens were fabricated using a DPL device (3Demax, DMG, Rapid Shape, Hamburg, Germany) with a 385-nm wavelength and a 100-µm layer thickness. Specimens were fabricated on a build platform using various build orientations, specifically including 0°, 90°, and an automatically generated 15° set by the layout software, as presented in Figure 1. After removing specimens from the platform, they were divided into three groups based on different post-washing times of 90, 120, and 150 s. Specimens underwent an efficient residue-removal process of being washed with 99% isopropyl alcohol within a predetermined cleaning time using a cleaning unit (3Dewash, DMG, Rapid Shape, Hamburg, Germany). Subsequently, specimens were air-dried at room temperature for 30 min and post-cured using a curing unit (3Decure, DMG, Rapid Shape, Hamburg, Germany) for 30 min to enhance the material’s final properties. Before the experiment, specimens were stored in distilled water at 37 °C for 24 h.

### 2.2. Accuracy of DLP-Fabricated Specimens

The accuracy of DLP-fabricated specimens was assessed by comparing dimensions of the fabricated bars to dimensions specified in the CAD. Additively manufactured permanent resin specimens were measured using digital calipers (150-mm Digital Calipers, with an accuracy of ±0.05 mm; Mitutoyo; Kanagawa, Japan). We assessed the length (25 mm), width (3 mm), and thickness (2 mm) of the specimens. At least five specimens were measured for each group, and three measurements were taken from different sections along the fabricated bars. To quantify the fabrication accuracy, discrepancies between the fabricated and CAD-designed specimens were calculated, and the average error was calculated based on all of the specimens.

### 2.3. Mechanical Properties

Disc-shaped specimens underwent polishing with 1000-grit SiC paper and were subsequently immersed in distilled water at 37 °C for 24 h. The VH of polished surfaces was measured using a microhardness tester (HM-101, Mitutoyo, Kawasaki, Japan) on a specimen build orientation of 0°. A load of 500 gf was applied to specimens for 10 s during the testing process. Each specimen was subjected to indentation testing at five different points, and the reported VH number (VHN) was the average of the indentation measurements taken at these locations.

A three-point bending test was performed to determine the flexural strength of specimens [23] using a universal testing machine (AGX-V, Shimadzu, Kyoto, Japan). Bar-shaped specimens (n = 10 per group) were positioned on two supporting points, with a 20-mm support span width. The height and width of the specimens at the midpoint were measured using a digital micrometer with an accuracy of 1 µm (MDC-250, Mitutoyo, Kawasaki, Japan). A crosshead speed of 1 mm/min was employed to apply the load at the center of the supporting points until fracture occurred. The flexural strength (FS) was determined by considering the fracture load and dimensions of the specimen, verified using digital calipers. Fracture loads obtained from the FS calculations were expressed in megapascal (MPa), using the following equation:

(1) $$FS=\frac{3FL}{\left({2BH}^{2}\right)}$$

where F is the load at the fracture point, L is the length of the support span, B is the width of the specimen, and H is the thickness of the specimen.

Then, the modulus (E) was determined in gigapascals (GPa) using the equation:

(2) $$E=\frac{{FL}^{3}}{{4BH}^{3}d}$$

where F is the maximum force, L is the distance between supports, B is the thickness of the specimen, H is its height, and d is the deflection (mm) when force F is applied. Additionally, the fractured surface morphology was examined using scanning electron microscopy (SEM) to gain insights into the fracture behavior and characteristics.

### 2.4. Degree of Conversion (DC)

Fourier-transform infrared (FTIR) spectra using the attenuated total reflection mode on an FT/IR-4000 type A instrument (Jasco Asia Portal; Tokyo, Japan) were employed to assess the DC of DLP-fabricated specimens (n = 5 per group) subjected to various cleaning times. The resin material in its liquid state and the post-cured DLP-fabricated specimens were analyzed. Ratios of absorbance peaks associated with the aliphatic double bond at 1637 cm^−1^ and the aromatic double bond at 1608 cm^−1^ were measured. The DC was calculated using the following equation [24]:

(3) $$DC\left(\%\right)=(1-\frac{{\left((|\frac{{Absorbance\;peak\;at\;1637\;cm}^{-1}}{{Absorbance\;peak\;at\;1608\;cm}^{-1}}\right))}_{DLP-fabricated\;specimens}}{{\left((|\frac{{Absorbance\;peak\;at\;1637\;cm}^{-1}}{{Absorbance\;peak\;at\;1608\;cm}^{-1}}\right))}_{in\;the\;liquid\;form}})\times 100$$

### 2.5. Water Sorption and Solubility

Disc-shaped specimens (n = 10 per group) were incubated in a desiccator at 37 °C for 22 h. Subsequently, they were transferred to another desiccator at 23 °C for 2 h, and their weights were measured. This process was repeated until the specimens lost < 0.1 mg, and this final weight was recorded as the baseline mass (m1). The diameter and thickness of a specimen were measured using a digital micrometer to determine its volume (V). Then, specimens were immersed in distilled water at 37 °C for 7 days. Subsequently, they were dried and weighed (m2). Following the weighing process, specimens were reconditioned to achieve a constant mass in a desiccator as described above. The stable mass was recorded as m3. The water sorption (Wsp) and solubility (Wsl) values were calculated using the following equations [25]:

(4) $$W_{sp}=\frac{m_{2}-m_{3}}{V}\;\mathrm{and}\;W_{sl}=\frac{m_{1}-m_{3}}{V}$$

### 2.6. Cytotoxicity Assay

Cytotoxicity was evaluated by a Cell Counting Kit (CCK)-8 assay with an NIH-3T3 fibroblast cell line (CRL-1658, ATCC, Taipei, Taiwan). Cells were cultured in minimum essential medium supplemented with 10% fetal bovine serum and 1% penicillin-streptomycin at 37 °C for 1 day. After detachment using trypsin, the cell suspension was adjusted to a density of 105 cells/mL. Five hundred microliters of a cell suspension was seeded onto specimens (n = 3) in a 24-well plate and incubated for 1, 3, 5, and 7 days at 37 °C in a CO_2_-humidified incubator. Following incubation, 10 μL of the CCK-8 solution (Sigma-Aldrich, St. Louis, MO, USA) was added to each well, and the plates were further incubated in a cell incubator for 3 h. The optical density (OD) value of each well, indicative of absorbance, was determined at 450 nm using an enzyme-linked immunosorbent assay (ELISA) reader (Epoch, Biotek Instruments, Highland Park, TX, USA).

The percentage cytotoxicity was calculated using the following equation:

(5) $$\mathrm{Cytotoxicity}\;(\%)\;=\;[\frac{{(OD}_{test\;specimen}-{OD}_{background})-{OD}_{negative\;control}}{{OD}_{positive\;control}-{OD}_{negative\;control}}]\times 100$$

where the background control was the medium only, the negative control group comprised DLP-fabricated specimens that did not undergo the cleaning process, and the positive control group consisted of cells cultured in tissue culture plates.

### 2.7. Statistical Analysis

Statistical analyses were performed using SPSS (IBM SPSS Statistics, v19.0; IBM, Armonk, NY, USA). A one-way analysis of variance (ANOVA) was used to study the effect of one factor (either build orientation or post-washing time) on the test properties. For the interaction of build orientation and post-washing time, a two-way ANOVA was used. Pairwise comparisons were performed using the post hoc Tukey honest significant difference (HSD) test with a significance level of α = 0.05. Results are presented as the mean ± standard deviation (SD), and *p* values were denoted by * (*p* < 0.05), ** (*p* < 0.01), and *** (*p* < 0.001).

## 3. Results

### 3.1. Dimensional Accuracy

**Figure 2:**
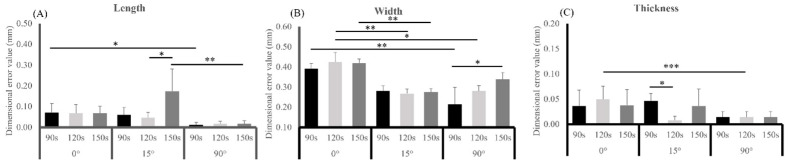
Means and standard deviations of dimensional error values (mm) for each fabricated specimen compared to the original STL in length, width, and thickness. Specimens were produced with three different printed orientations (0°, 15°, and 90°) and three different post-washing times (90, 120, and 150 s). Results are presented as follows: (**A**) length, (**B**) width, and (**C**) thickness of the test. All statistical differences are denoted by * (*p* < 0.05), ** (*p* < 0.01), and *** (*p* < 0.001).

Figure 2 shows the accuracy of fabricated specimens as related to build orientations and post-washing times. Dimensions of fabricated specimens were greater than those of the original STL regardless of the measured direction. The smallest measurement error among the specimens was in thickness, followed by length, while the largest error was observed in width (with discrepancies ranging 0.21–0.42 mm). The two-way ANOVA revealed that both the build orientation (*p* < 0.001 for length and width; *p* < 0.007 for thickness) and post-washing time (*p* < 0.029 for length and *p* < 0.006 for width), and their interaction (*p* < 0.009 for length and *p* < 0.010 for width) significantly influenced dimensions, as detailed in Table 1. Consequently, it was confirmed that the build orientation affected dimensions in all three directions.

### 3.2. Mechanical Properties

**Figure 3:**
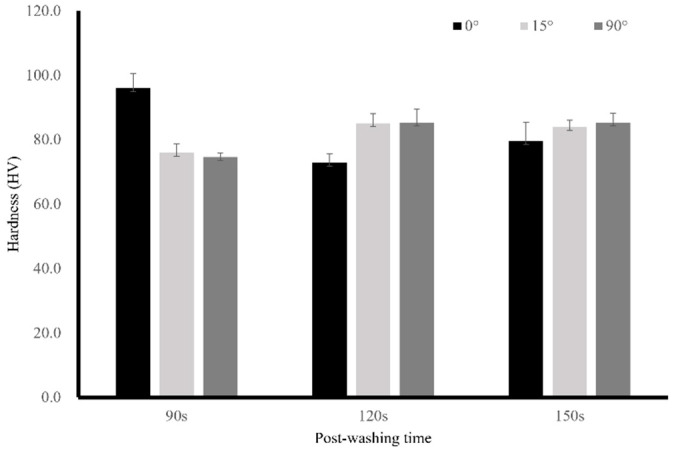
Means and standard deviations of Vickers hardness value (VHV) measurements of specimens with three different post-washing times (90, 120, and 150 s).

VHVs of DLP-fabricated permanent resin specimens with different post-washing times are displayed in Figure 3. There was a positive trend observed with increasing post-washing time correlated with an increase in VH; however, the one-way ANOVA revealed no statistical differences among post-washing times.

**Figure 4:**
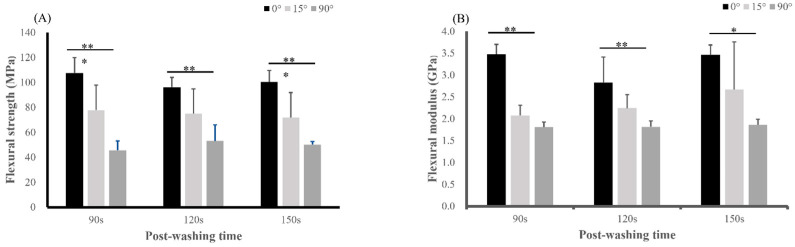
Means and standard deviations of a three-point bending test with three different printed orientations (0°, 15°, and 90°) and three different post-washing times (90, 120, and 150 s). Results are presented as follows: (**A**) flexural stress and (**B)** flexural modulus. All statistical differences are denoted by * (*p* < 0.05), and ** (*p* < 0.01).

Means and SDs of obtained three-point bending strengths and elastic moduli are respectively illustrated in Figure 4A,B. A two-way ANOVA conducted on flexural strength revealed that build orientation had a highly significant effect (*p* < 0.001), indicating that the direction in which the specimens were built is a critical determinant of their resistance to bending forces. Post-washing time also exhibited a significant (*p* = 0.024), affecting the mechanical properties of the specimens, although not as markedly as build orientation. Additionally, a significant interaction between build orientation and post-washing time was observed (*p* = 0.009), indicating a combined effect on the overall flexural strength behavior of specimens. In terms of the flexural modulus, build orientation demonstrated a significant (*p* < 0.001) effect, as did post-washing time (*p* = 0.006). Moreover, the interaction between build orientation and post-washing time was significant (*p* = 0.010), consistent with the interaction observed for flexural strength. Different build orientations alter interlayer exposure and solvent penetration pathways, thereby modulating the effect of post-washing on mechanical properties.

**Figure 5:**
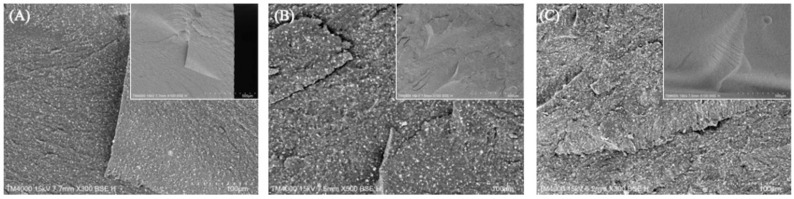
Representative SEM images of fracture surfaces of DLP-fabricated permanent resin specimens with various post-washing times: (**A**) 90 s, (**B**) 120 s, and (**C**) 150 s.

Therefore, we conducted subsequent DLP-fabricated permanent resin experiments using 0° orientation. In addition, Figure 5A–C present representative SEM images of fracture surfaces of DLP-fabricated permanent resin specimens with various post-washing times. Images reveal a progressive increase in surface voids and grooves correlated with extended post-washing times. This trend indicated that prolonged post-washing can alter the surface morphology, potentially affecting the mechanical integrity and performance of the resin.

### 3.3. Degree of Conversion (DC)

**Figure 6:**
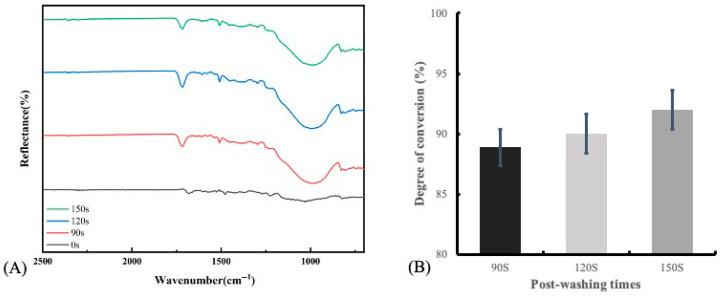
(**A**) FTIR spectra of DLP-fabricated permanent resin specimens with three different post-washing times (90, 120, and 150 s). Spectra of specimens without post-curing (control group) are also displayed. (**B**) The degree of conversion (DC) of DLP-fabricated specimens with different post-washing times.

FTIR spectra of DLP-fabricated permanent resin specimens with different post-washing times are displayed in Figure 6A. The spectrum of the specimen without post-curing exhibited characteristic peaks corresponding to methacrylate resins, including the ester C=O stretching band around 1720 cm^−1^, CH_2_ bending near 1450 cm^−1^, and C–O–C stretching bands in the 1000–1200 cm^−1^ region. In contrast, the peak of aromatic C=C stretching near 1606 cm^−1^ was not prominent. After post-curing and post-washing, the ester C=O stretching band shifted slightly to higher wavenumbers, consistent with the conversion from monomer to polymer. Moreover, the intensity of the aromatic C=C stretching peak increased progressively with longer post-washing times. Compared to uncured specimens, the DC of the 3D-printed permanent resin specimens increased with post-washing time; however, the difference was not statistically significant (*p* = 0.214), as shown in Figure 6B.

### 3.4. Water Sorption and Solubility

**Figure 7:**
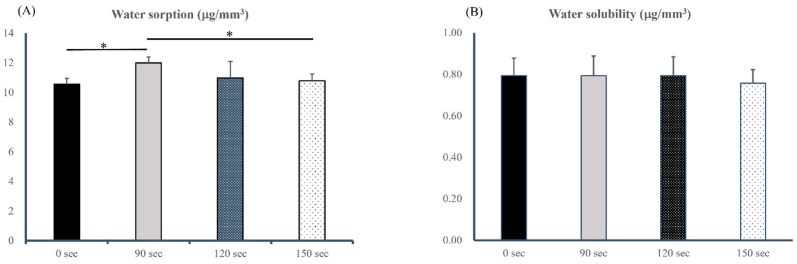
Water sorption and water solubility of DLP-fabricated permanent resin specimens with three different post-washing times (90, 120, and 150 s). Results are presented as follows: water sorption (**A**) and water solubility (**B**). All statistical differences are denoted by * (*p* < 0.05).

Results of water sorption and solubility are presented in Figure 7A,B. The highest sorption value (11.99 ± 0.39 μg/mm^3^) was observed for DLP-fabricated permanent resin with a post-washing time of 90 s, while the lowest value of 10.56 ± 0.39 μg/mm^3^ was recorded for the 3D-printed permanent resin without post-washing, as shown in Figure 6A. A one-way ANOVA conducted on Wsp values revealed a significant effect of post-washing time (*p* = 0.018). This finding suggests that the post-washing time significantly affected the Wsp. Values of water solubility ranged 0.75–0.79 μg/mm^3^, as shown in Figure 6B. Conversely, post-washing time exhibited no statistically significant effect on water solubility (*p* = 0.888). This implies that the post-washing time did not significantly alter the Wsl.

### 3.5. Cytotoxicity

**Figure 8:**
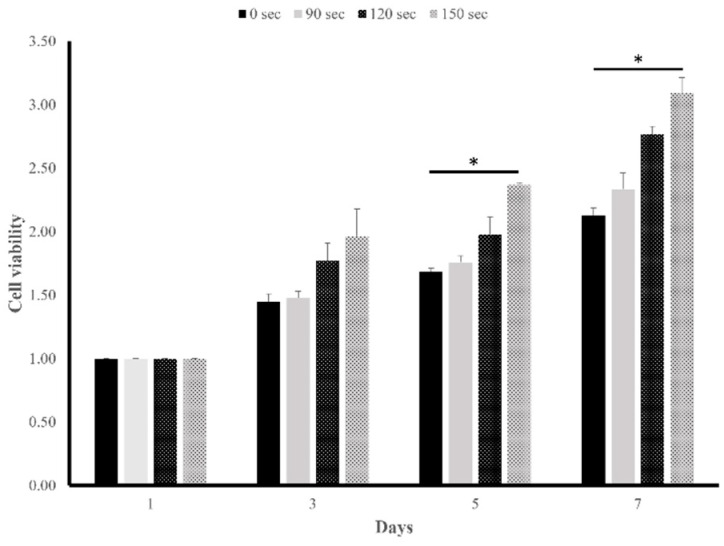
Means and standard deviations of cytotoxicity results of NIH-3T3 cells after 7 days of incubation in extracts with three different post-washing times (90, 120, and 150 s). * Statistically significant difference between specimens (*p* < 0.05).

Figure 8 shows cytotoxicity results measured by an MTT assay, revealing that there was an increase in cell viability with an extension of post-washing times and days. The 150-s group registered the highest cell viability, in contrast to the 0-s group which showed the lowest (as shown in Figure 7A). There was a significant difference in post-washing times between the 150- and 0-s groups on days 3, 5, and 7, with respective *p* values of 0.028, 0.013, and 0.013. Under optimized culture or experimental conditions, high OD values were indicative of increased cell viability. However, according to the ISO 10993-5 standard [26], all of the DLP-fabricated resin materials showed over 70% relative viability, indicating that the DLP-fabricated resin material was non-toxic and suitable for oral environments.

## 4. Discussion

There is no doubt that DLP-fabricated materials currently have great potential in clinical dentistry, particularly when considering the permanent crown material investigated in this study. The ability to manufacture crowns and bridges in under 30 min not only reduces patient appointment duration but also augments overall clinic efficiency. This has the potential to increase clinic productivity and provide practical, rapid, and precise restorative solutions. Therefore, the provision of cost-effective and readily available 3D-printed restorative materials warrants exploration, with the aim of evaluating whether they have the appropriate physical and mechanical properties. In the present study, we found a significant influence of build orientation on mechanical properties and accuracy of DLP-fabricated permanent resin. Hence, the first null hypothesis was partially rejected. Results demonstrated that the post-washing time did not have a significant impact on flexural strength, HV, DC, water sorption and solubility, or cell viability. Therefore, the second null hypothesis was partially rejected. In the present study, we found a significant influence of build orientation on dimensional accuracy and flexural strength.

Interestingly, the accuracy of DLP-fabricated materials exhibited notable variations depending on both the build orientation and specific structural area under evaluation. DLP-fabricated specimens demonstrated a consistent dimensional accuracy of <0.2 mm, with the 90° orientation achieving the highest precision among all tested configurations. As for the width of DLP-fabricated specimens, significant discrepancies were observed in cases where orientations of 0°, 15°, or 90° were used, with the most substantial deviations being particularly evident in specimens fabricated at a 0° orientation, which was consistent with previous research [27].

With each increase in the build-orientation angle, the flexural strength decreased. However, there was no significant difference in flexural strengths at different post-washing times, a result similar to that of the VH. In three-point bending tests of dental resin materials, fractures were one of the main reasons for clinical failure of definitive restorations [28]. In the present study, the minimum flexural strength at a 0° orientation exceeded 100 MPa, surpassing that standardized by ISO 4049–2019 for resin-based restorations in dentistry [28]. Although flexural strengths at 15° and 90° orientations were relatively lower, they still exceeded the physical requirement of 50 MPa for polymer-based crown and bridge materials in ISO 10477:2020 [22]. However, another study found that the 90° orientation was optimal but had no effect on the mechanical performance [27], which contrasts with results of the present study. Regarding VH, which is the ability to resist plastic deformation, in prior research, the average VH of DLP-fabricated resin crown and bridge materials was reported to be 20.9 kgf/mm^2^ [29]. However, in the present study, when printed at a 0° orientation and subjected to various post-washing times, no significant difference in VH values was observed among the different groups. Average VH values for all groups exceeded 70 kgf/mm^2^, a notably higher value compared to findings reported in previous research. These results suggest enhanced resistance to plastic deformation in the materials utilized in this study, potentially indicating improved mechanical properties. Additionally, due to the lower elastic modulus of the resin compared to dentin, this could potentially result in stress accumulation within the remaining tooth structure. The elastic modulus, a measure of an object’s or substance’s resistance to elastic deformation, remained unchanged regardless of post-washing durations of 90, 120, or 150 s, demonstrating consistent values across these conditions. This also aligns with the mechanical properties provided by the manufacturer.

The water absorption of dental resin materials depends on the hydrophilicity of the resin matrix and filler composition [30], and exposure to an oral saliva environment can lead to material degradation and erosion [31]. In the present study, we observed that water absorption did not significantly increase with prolonged post-washing times. Even with extended washing times, there was no reduction in the polymer matrix density, which led to no increase in water absorption, which could have potentially compromised its performance. Therefore, the tested DLP-fabricated permanent crown material may be considered suitable for clinical applications.

Results of the cell viability test demonstrated significant enhancement in cell viability and a reduction in cytotoxicity, particularly when extending the washing time beyond 150 s, compared to the control group after the 5th and 7th days (Figure 8). This improvement may be attributed to the use of 99% isopropyl alcohol washing before post-curing, which effectively reduced unreacted monomer residues on the surface of the DLP-fabricated permanent crown material. The post-washing process likely minimized the impact on cell vitality by reducing monomer residues on the unpolymerized layer that remained after post-curing.

Based on the safety data sheet, the resin contains multifunctional dimethacrylate cross-linkers, low-molecular-weight monomers, and phosphine oxide photoinitiators. These components account for the high strength but brittle fracture behavior, the influence of residual monomers on water sorption and cytocompatibility, and the dependence of DC on post-curing. Our results further indicated that longer post-washing times were associated with improved cell viability and higher DC. Prolonged washing more effectively removes unpolymerized (meth)acrylate monomers and photoinitiator residues, both of which are known to compromise cytocompatibility. Given that the resin composition includes multifunctional dimethacrylates and phosphine oxide initiators, insufficient removal of these residual components may result in incomplete polymerization and increased cytotoxicity. In contrast, extended washing improves resin surface quality and reduces the amount of leachable compounds, thereby contributing to both higher DC and enhanced cellular responses.

One limitation of the present study is that only one type of DLP-fabricated permanent crown material was used. Quantification of the mechanical, characteristic, and biological properties was based on standardized specimens. Different types of printing equipment and resins can yield various results, which could lead to different outcomes for final fabricated prostheses. Furthermore, the current study did not simulate chewing loads in the oral environment or fatigue testing. Therefore, further investigations considering the importance of these factors may impact the material characteristics of DLP-fabricated permanent restorations. Nonetheless, the present study demonstrated that 3D-printed permanent crown material indeed enhanced the mechanical strength, water absorption and solubility, and biocompatibility. As there is limited research comparing the performances of various DLP-fabricated permanent resins, our study can serve as a valuable foundation for clinical practices related to DLP-fabricated permanent resin restorations. Another limitation concerns the ATR-FTIR measurements. Since ATR spectra are influenced by refractive index differences does not strictly apply, the absolute values of conversion may be affected. However, as all specimens were measured under identical conditions, the relative comparisons remain reliable and provide meaningful insights.

## 5. Conclusions

In this in vitro study, effects of build orientations and post-washing times on DLP-fabricated permanent crown material were explored. Findings revealed that the build orientation of the resin significantly impacted its dimensional accuracy and flexural strength. In contrast, post-washing time exhibited no notable differences in the degree of conversion, hardness, water absorption, or solubility. Notably, an extended post-washing time markedly improved cell viability and reduced cytotoxicity, without adversely affecting the physical or mechanical properties of DLP-fabricated permanent dental crown materials.

## Figures and Tables

**Table 1:** Results of a two-way ANOVA of the effects of build orientation and post-washing time on length, width, and thickness of DLP-fabricated specimens.

Dimensional Direction	Source	Type III Sum Squares	df	Mean Square	F	*p*
Length	Build orientation	0.049	2	0.025	11.641	<0.001 ***
	Post-washing time	0.016	2	0.008	3.923	0.029 *
	Build orientation × Post-washing time	0.033	4	0.008	3.935	0.009 **
Width	Build orientation	0.186	2	0.093	60.417	<0.001 ***
	Post-washing time	0.018	2	0.009	5.832	0.006 **
	Build orientation × Post-washing time	0.024	4	0.006	3.890	0.010 **
Thickness	Build orientation	0.006	2	0.003	5.696	0.007 **
	Post-washing time	0.000	2	0.000	0.501	0.610
	Build orientation × Post-washing time	0.004	4	0.001	1.991	0.117

## Data Availability

The original contributions presented in this study are included in the article. Further inquiries can be directed to the corresponding authors.

## Abbreviations

DLP	Digital light processing
CAD/CAM	Computer-aided design/computer-aided manufacturing
SLA	Stereolithography apparatus
